# Carvajal Syndrome- A Variant of Naxos Disease: A Case Report

**DOI:** 10.31729/jnma.7102

**Published:** 2022-02-28

**Authors:** Krishna Deo Mandal, Pun Narayan Shrestha, Anjila Ghimire, Prakash Joshi, Sumit Agrawal, Prapti Shrestha

**Affiliations:** 1 Kanti Children's Hospital, Maharajgunj, Kathmandu, Nepal

**Keywords:** *cardiomyopathy*, *case report*, *palmoplantar keratoderma*, *rare disease*

## Abstract

Carvajal syndrome is a rare variant of Naxos disease, a recessive mutation of the desmoplakin gene characterized by presence of woolly hair, palmoplantar keratoderma and dilated cardiomyopathy, mainly left ventricular involvement. The main clinical complication is progressive heart disease which may lead to heart failure and sudden cardiac death in childhood and adolescence. Cardiomyopathy is diagnosed by Task Force Criteria. The goal of treatment is to prevent sudden cardiac death by lifestyle modification and regular clinical monitoring with pharmacotherapy. We report a nine years female who had skin and hair abnormality and was admitted with features of heart failure. She was clinically diagnosed as Carvajal syndrome, an under-recognized cardio cutaneous manifestation in children. Clinicians should be aware, if any child present with keratoderma of palm and soles with woolly hair since birth should evaluate for cardiomyopathy. Genetic tests should be done whenever available, for confirming the diagnosis and counseling.

## INTRODUCTION

Carvajal syndrome is a cardiocutaneous condition characterized by palmoplantar keratoderma, woolly hair, and cardiomyopathy. Its inheritance follows an autosomal recessive pattern of desmoplakin gene.^1^ The symptoms manifest over time; woolly hair is present from birth while palmoplantar keratoderma occurs after infancy particularly at pressure sites. It associated dilated cardiomyopathy (DCM) which is usually asymptomatic at an early age.^2^ The main clinical complication is progressive heart disease due to fibrosis of myocardium which may lead to heart failure and sudden cardiac death in childhood and adolescence.^3^

We report a nine years female with heart failure and clinically diagnosed with Carvajal syndrome.

## CASE REPORT

**Figure 1:**
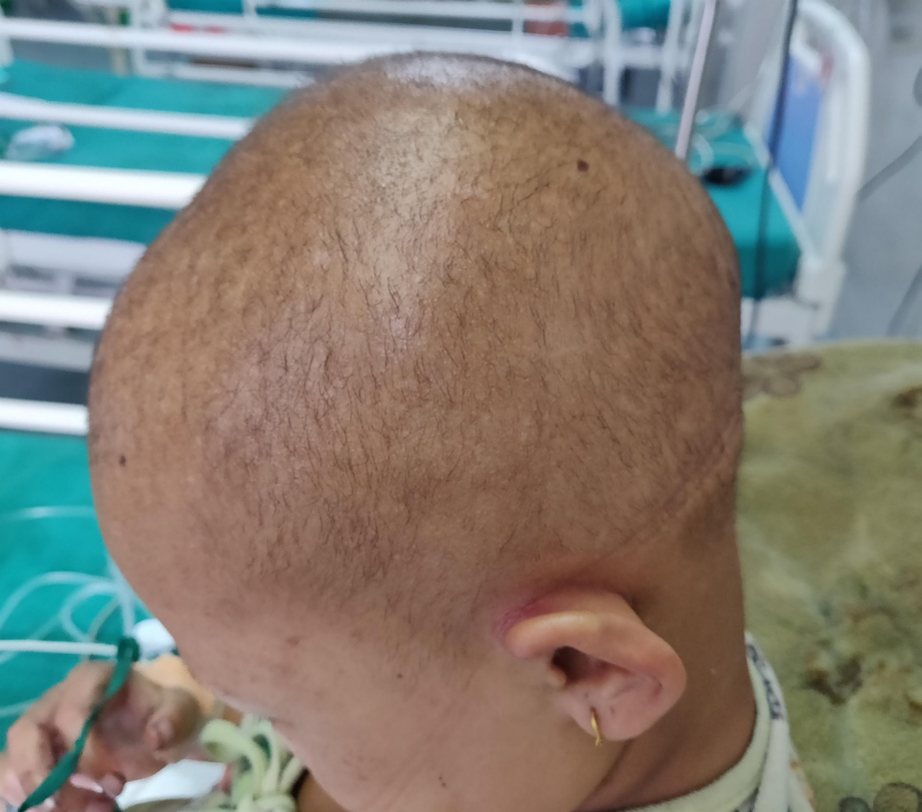
Woolly, thin, and scanty hair on the scalp.

A nine years female was admitted to our hospital with complaints of swelling of the body associated with difficulty in breathing for five days. She had pedal edema and facial puffiness associated with dyspnea of the same duration. She has had woolly and curly hair since birth. Cutaneous manifestations like the peeling of the skin, multiple keratotic papules, and plaque over palms and soles appeared at nine months of age. Her past medical history was free of other systemic diseases or allergies except for dermatitis of hands and feet, for which she had multiple visits at a local hospital and received conservative treatment. She was born via non-consanguineous marriage; her family history was insignificant. She has a younger brother who is free of disease. Clinically at the time of presentation, she was tachypneic with a respiratory rate of 48/min, pulse rate 112/min, and blood pressure of 80/40 mm of Hg. Oxygen saturation was 92% in room air. She had facial puffiness with raised Jugular venous pressure, bilateral pedal edema, thin and scanty hairs on the scalp (Figure 1).

**Figure 2:**
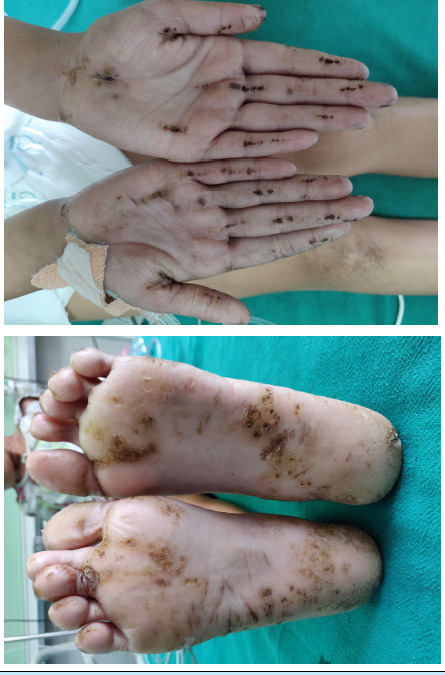
A) Keratoderma of palms; B) Keratoderma of soles.

Also, the patient had keratotic skin on palms and soles (Figure 2 A, B).

**Figure 3:**
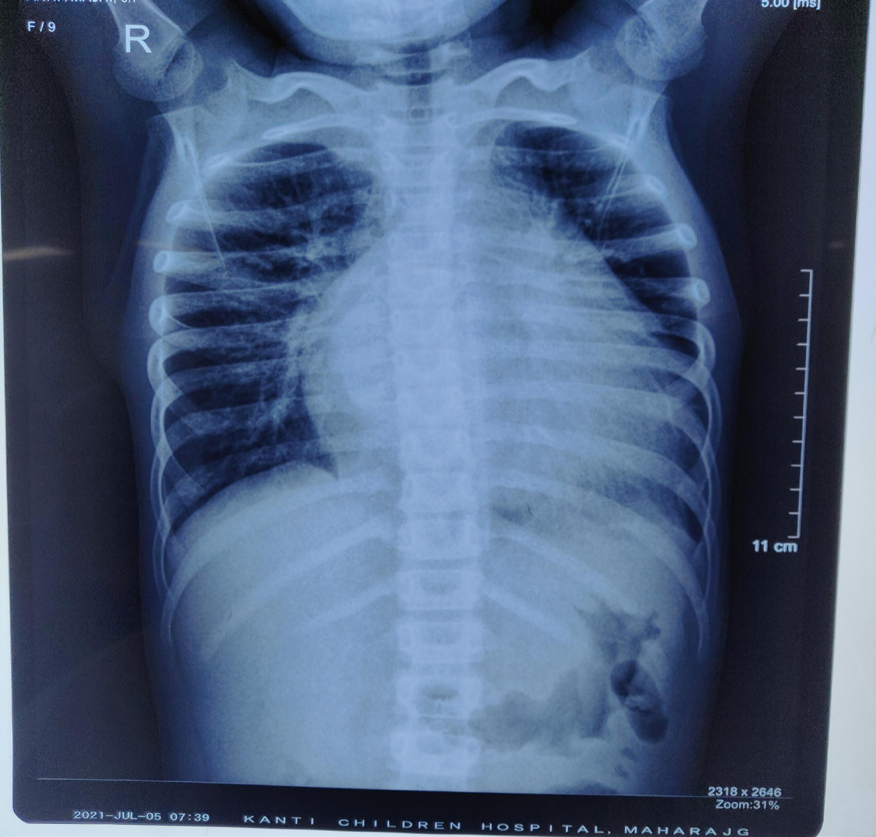
Chest x-ray showed cardiomegaly.

Cardiovascular examination revealed a hyperdynamic apical impulse with grade II systolic murmur over mitral and tricuspid areas with normal 1^st^ and 2^nd^ heart sounds. Subcostal retractions with bilateral basal crepitations were found on chest examination. There was hepatomegaly. The liver was palpable 4cm below the right subcostal margin with a span of 14cm; hepatojugular reflux was positive. Blood investigations showed elevated Troponin-I 10.023ng/ml (reference normal: <0.012) and creatine kinase myocardial band (CPK-MB) 51 International Units per liter (IU/L) (normal reference: 5-25IU/L). Kidney, liver function tests were within normal range. Arterial blood gas (ABG) revealed metabolic acidosis. Chest x-ray showed cardiomegaly (Figure 3).

Electrocardiography (ECG) as low voltage with sinus tachycardia and premature ventricular complexes, T wave abnormality in pericardial chest lead (Figure 4).

**Figure 4:**
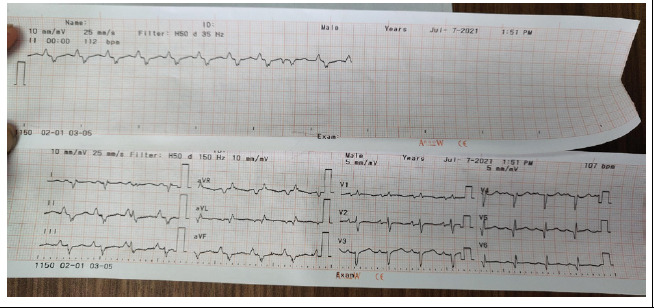
First ECG done at time of admission showed sinus tachycardia with low voltage, premature ventricular beats with T wave abnormality negative QRS in II, III, and AVF.

**Figure 4B:**
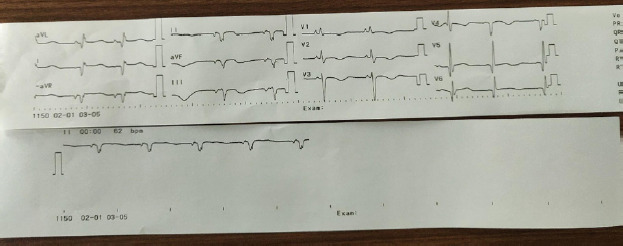
Second ECG on the 5th day showed an inverted T wave in v1-v4.

**Figure 5:**
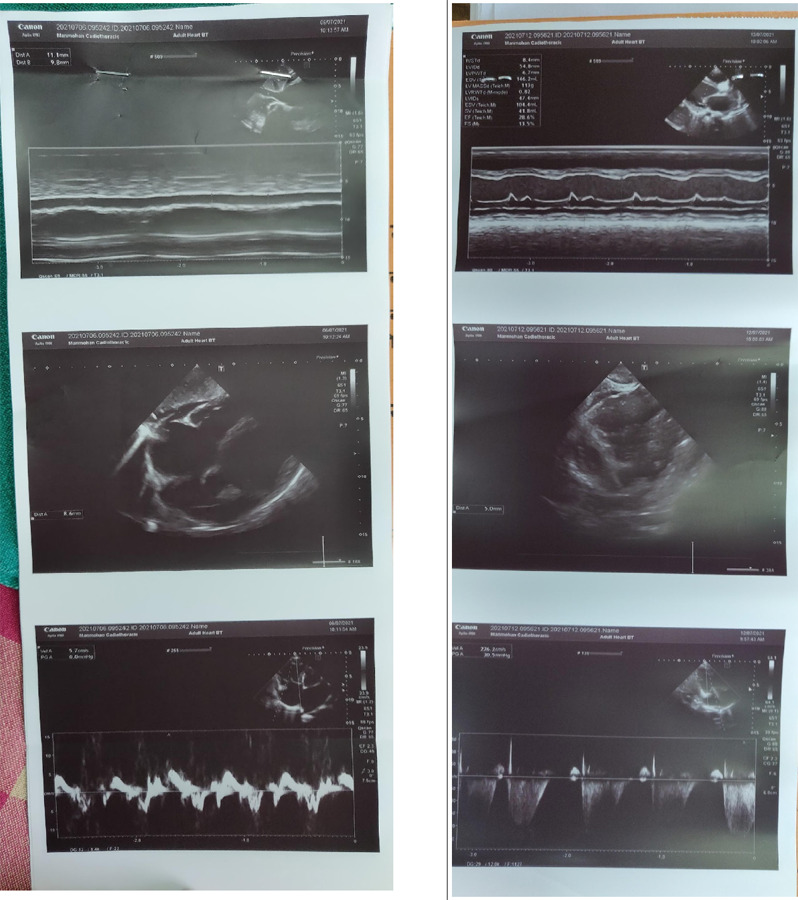
ECHO showed dilated all chambers more toward the left ventricles with ejection fraction 20%, regurgitation of mitral and tricuspid valves.

Echocardiography (ECHO) revealed severe biventricular dysfunction, dilated left ventricle (LV) with global hypokinesia, ejection fraction 20%, moderate mitral and mild tricuspid regurgitation (gradient 26 mm of Hg), mild pulmonary arterial hypertension (36 mm of Hg), and minimal pericardial effusion (Figure 5).

Supportive management was done with diuretics, Angiotensin-converting enzyme (ACE) inhibitors (Enalapril), inotropes (Dobutamine), and antibiotics. In response to treatment, pedal and pulmonary edema resolved within two days. Repeat ECHO & ECG was done on the fifth day of hospital stay. ECG showed inverted T-wave V1-V4 (Figure 4) and ECHO showed LV ejection fraction 20-25%. After maintaining hemodynamic stability she was shifted to a general ward and discharged on diuretics, enalapril, and Aspirin. For one-month, follow-up echocardiography showed left ventricular thrombus formation. She was started on anticoagulant (Warfarin) and later beta-blocker (Metoprolol) was added. She was finally continued with Warfarin, Metoprolol, diuretics, and emollient ointment. Hence, with all these clinical findings the patient was diagnosed with Carvajal syndrome and managed symptomatically.

## DISCUSSION

Carvajal syndrome is a rare variant of Naxos disease, first reported from the Greek island of Naxos.^4^ Genetic analysis showed that both diseases are related to genes encoding the cell adhesion desmosomal protein: a recessive mutation of plakoglobin gene causing Naxos disease and desmoplakin gene causing Carvajal syndrome.^1,3,4^ They share a characteristic triad of cardiomyopathy, palmoplantar keratosis, and woolly/ curly hair. The cardiomyopathy in Naxos disease is characterized by right-dominant ventricular dilatation, hypokinesia, and tachyarrhythmia; traditionally called arrhythmogenic right ventricular cardiomyopathy (ARVC).^3,4^ Diagnosis is often established in adolescence with arrhythmia, syncope, or ventricular tachyarrhythmia (VT). In contrast, Carvajal syndrome predominantly involves the left ventricle, resembling dilated cardiomyopathy, and presents at an early age of life with heart failure.^1-4^ The present case had dilated LV with global hypokinesia as well as features of heart failure, consistent with Carvajal syndrome.

Furthermore, ARVC (Naxos disease) can progress to left ventricular involvement and overlap with dilated cardiomyopathy thus creating difficulty in identifying the two syndromes.^2,4^ However, Revised Task Force Criteria brings about uniformity in recognition and diagnosis of ARVC.^5^ Definitive diagnosis requires 2 major criteria, or 1 major and 2 minor criteria or 4 minor criteria. Our patient has 3 major criteria: 2D ECHO showed severe biventricular dysfunction, on ECG repolarization abnormality; inverted T wave in V1-V4, arrhythmia; negative QRS in II, III, and AVF, and one minor criteria of ventricular ectopy. Hence, our patient fulfills the criteria for ARVC. According to a recent literature review if cardiomyopathy (ARVC) is spread over both ventricles, even more, severe changes on the left ventricles with cutaneous manifestation is referred to as Carvajal syndrome.^3^

Mutation in desmosomal protein led to an abnormality in the cell-to-cell adhesion, disrupt tissue integrity and cause keratoderma in the skin.^3,4^ Inflammatory damage to myocytes is followed by repair with fibrous or fibrofatty tissue in the myocardium. This may cause electrical dysfunction as arrhythmias and contractile dysfunction as heart failure.^6^ Stress-induced tissue loss at a pressure point in limbs when a child starts using hands and feet explain the occurrence of palmoplantar keratoderma. Woolly hair is present since birth; it can be hereditary or localized nonhereditary.

The diagnosis of the disease is made using history, a combination of clinical, electrocardiography, radiology, and histopathological feature as described by task force criteria.^5^ The classical ECG finding including, microvoltage, premature ventricular contraction, precordial T-wave inversion/flattening in V1-V3, gradually extended to inferior leads, consistent with ECG finding in the present case.^1,3,6^ Structure and functional abnormality of heart detected by echocardiography include biventricular dilation, mostly left ventricle with global or regional hypokinesia, thrombus formation, and reduced left ventricular ejection fraction.^3,5,6^ Reduction in ejection fraction up to 21% has been reported.^7^ Similarly in the current case the ejection fraction was 20% with LV thrombus formation.

Cardiac magnetic resonance has several advantages over echocardiography; it analyzes the heart tissue, epicardium, myocardium, infiltration of other cells-fibrosis and/or adipocytes.^8^ However, it may lead to false interpretation about the tissue infiltration.^3^ Biopsy is a method for diagnosis but it has high specificity and low sensitivity as pathological changes within the heart are not equally distributed.^3^ Due to unavailability, these tests were not done in our index. The endomyocardial biopsy and genetic test may be done in selected cases that meet the task force criteria to confirm the diagnosis.^9^ However, a negative genetic test does not exclude the disease.^7^ Thus, it highlights that diagnosis can be made with the help of ECG and echo findings in a patient with classical skin and hair abnormality where genetic Lab is not available. However, genetic testing seems essential for confirmation and counseling for future pregnancy.^10^

Lifestyle modification, health education, and regular clinical monitoring are the primary goals of treatment to prevent sudden cardiac death due to arrhythmia and severe heart failure.^3,4^ Antiarrhythmic agents like Amiodarone and Carvedilol have been widely used to treat VT and ventricular fibrillation. Diuretics and ACE inhibitors are commonly used for the treatment of heart failure and antithrombotic prophylaxis is given to prevent intracardiac thrombus formation.^3,6,9^ Patients with a history of cardiac arrest, recurrent or unstable VT not well controlled with pharmacological agents are candidates for automated implantable cardioverter-defibrillator.^3,8,9^

Clinicians should be aware of any child present with keratoderma of palm and soles with woolly hair since birth should be evaluated for cardiomyopathy. Such a patient is at high risk of sudden cardiac death due to arrhythmia or heart failure. ECG, echocardiography provides more evidence to support predominant left ventricular cardiomyopathy, a Carvajal syndrome, an under-recognized cardiocutaneous manifestation. Genetic tests should be done whenever available, for confirming the diagnosis and counseling.
